# Role of tyrosine kinase inhibitor in chronic myeloid leukemia patients with SARS-CoV-2 infection: A narrative Review

**DOI:** 10.1097/MD.0000000000029660

**Published:** 2022-06-30

**Authors:** Muhammad Asif, Muhammad Amir, Abrar Hussain, Niaz M. Achakzai, Peter Natesan Pushparaj, Mahmood Rasool

**Affiliations:** a Department of Biotechnology, BUITEMS, Quetta, Pakistan; b Office of Research Innovation and Commercialization, BUITEMS, Quetta, Pakistan; c Department of Molecular Biology, City Medical Complex, Kabul, Afghanistan; d Department of Molecular Biology, DNA section, Legal Medicine Directorate, Ministry of Public Health, Kabul, Afghanistan; e Center of Excellence in Genomic Medicine Research, Faculty of Applied Medical Sciences, King Abdulaziz University, Jeddah, Saudi Arabia; f Department of Medical Laboratory Technology, Faculty of Applied Medical Sciences, King Abdulaziz University, Jeddah, Saudi Arabia.

**Keywords:** bronchoalveolar epithelial cells, chronic myelogenous leukemia, SARS-CoV-2, tyrosine kinase inhibitor

## Abstract

Severe acute respiratory syndrome (SARS) caused by a novel coronavirus-2 (CoV-2), also known as COVID-19, has spread rapidly worldwide since it is recognized as a public health emergency and has now been declared a pandemic on March 11, 2020, by the World Health Organization.

The genome of SARS-CoV-2 comprises a single-stranded positive-sense RNA approximately 27 to 30 kb in size. The virus is transmitted through droplets from humans to humans. Infection with the SARS virus varies from asymptomatic to lethal, such as fever, cough, sore throat, and headache, but in severe cases, pneumonia and acute respiratory distress syndrome.

Recently, no specific and effective treatment has been recommended for patients infected with the SARS virus. However, several options can be investigated to control SARS-CoV-2 infection, including monoclonal antibodies, interferons, therapeutic vaccines, and molecular-based targeted drugs.

In the current review, we focus on tyrosine kinase inhibitor management and their protective role in SARS-CoV-2 patients with chronic myelogenous leukemia.

## 1. Introduction

The SARS-CoV (betacoronavirus, lineage B) in human mainly affects the upper respiratory tract and gastrointestinal tract typically related to fever,myalgia, headache, malaise, and chills, followed by a nonproductive cough, dyspnea.^[1]^ It is a severe acute respiratory syndrome coronavirus (SARS-CoV) that began in southern China during 2002, Guangdong, which caused human infections to death in more than 8000 and 775, respectively expended in 37 countries.^[2,3]^ After a decade in Saudi Arabia in, 2012 an additional epidemic was witnessed in the form of Middle East respiratory syndrome coronavirus (MERS-CoV).^[4]^ In addition, more than 2500 confirmed laboratory cases of infections and approximately 170 deaths while <80% of cases were reported from Saudi Arabia.^[5]^

In December 2019, several pneumonia cases due to an unidentified etiology were reported in Wuhan City, Hubei Province, China. Epidemiological investigations revealed that these patients had viral pneumonia associated with Seafood Wholesale Market Huanan.^[6,7]^ Initially, investigators believed that individuals who were exposed to the market and developed viral pneumonia suggested animal-to-human SARS-CoV-2 transmission. However, those who were diagnosed very recently and had no direct experience of market indorsing that spread between humans.^[8,9]^

Later, the infective agent responsible for a respiratory infection is known as novel severe acute respiratory syndrome coronavirus-2 (SARS-CoV-2).^[10]^ This virus emerged very recently in 2019 and was named coronavirus infectious disease-19 (COVID-19).^[11]^ This novel coronavirus (nCoV) has spread in more than 200 countries. As for the weekly World Health Organization (WHO) update on August 16, 2020, the aggregate confirmed cases including deaths worldwide were about 21.2 million and 761,000, respectively,.^[12]^ Due to concerning major public health on March 11, 2020, WHO professed the coronavirus transmission as a pandemic.^[13]^

In the current review, we focused on tyrosine kinase (TK) inhibitors’ management and their protective role in CML patients with SARS-CoV-2.

### 1.1. Coronavirus: taxonomy and morphology

Coronaviruses are enveloped, positive sense, single-stranded RNA, with a genome size of approximately 26 to 30 kb and (62–140 nm) in diameter and a largest solitary group of viruses belonging to the family *Coronaviridae* that are further categorized into 2 subfamilies (Letovirinae and Orthocoronavirinae), which consist of 4 genera: alpha, beta, delta, and gamma coronavirus (α, β, δ, γ-CoV), respectively.^[14–16]^ Corona in Latin means (crown) attributed to the appearance of spike glycoproteins is pointed like structures on their envelope under the electron microscope.^[17]^

### 1.2. SARS-CoV-2: genomic organization

Principally, investigation of the novel coronavirus-2019 known to be largest in the 2 RNA genomes encompasses 2 untranscribed regions (5’ and 3’ UTRs), 11 open reading frames (ORFs), which translate into 27 proteins and 4 structural proteins, which include S (spike) glycoprotein, E (envelope), M (matrix), and N (nucleocapsid).^[18,19]^ The 6 supplemental proteins that encode are (orf3a, orf6, orf7a, orf7b, orf8, and orf10).^[20,21]^

### 1.3. SARS-CoV-2: phylogenetic analysis

Phylogenetic-based studies have shown that SARS-CoV-2 forms a distinctive genetic lineage and shares more than 96% homology with bat coronavirus (RaTG13), confirming its zoonotic origin.^[22,23]^ To determine the genetic relationship between the 92 entire genome nucleotide, acute respiratory disease sequences were selected and retrieved from the GenBank NCBI database, as they were representative of the diverse geographical locations where minor and major respiratory disease outbreaks had previously been recorded in these regions..

Multiple sequence alignment (MSA) and phylogenetic reconstruction based on the complete genome nucleotide sequences were performed using the MAFFT version 7 program.^[24]^ A Jukes-Cantor (JC) model of nucleotide substitution was used to construct a neighbor-joining (NJ) tree. The robustness of the phylogeny was evaluated by resampling 1000 bootstrap replicates. The tree was used in MEGA X for color.^[25]^

**Figure 1.**
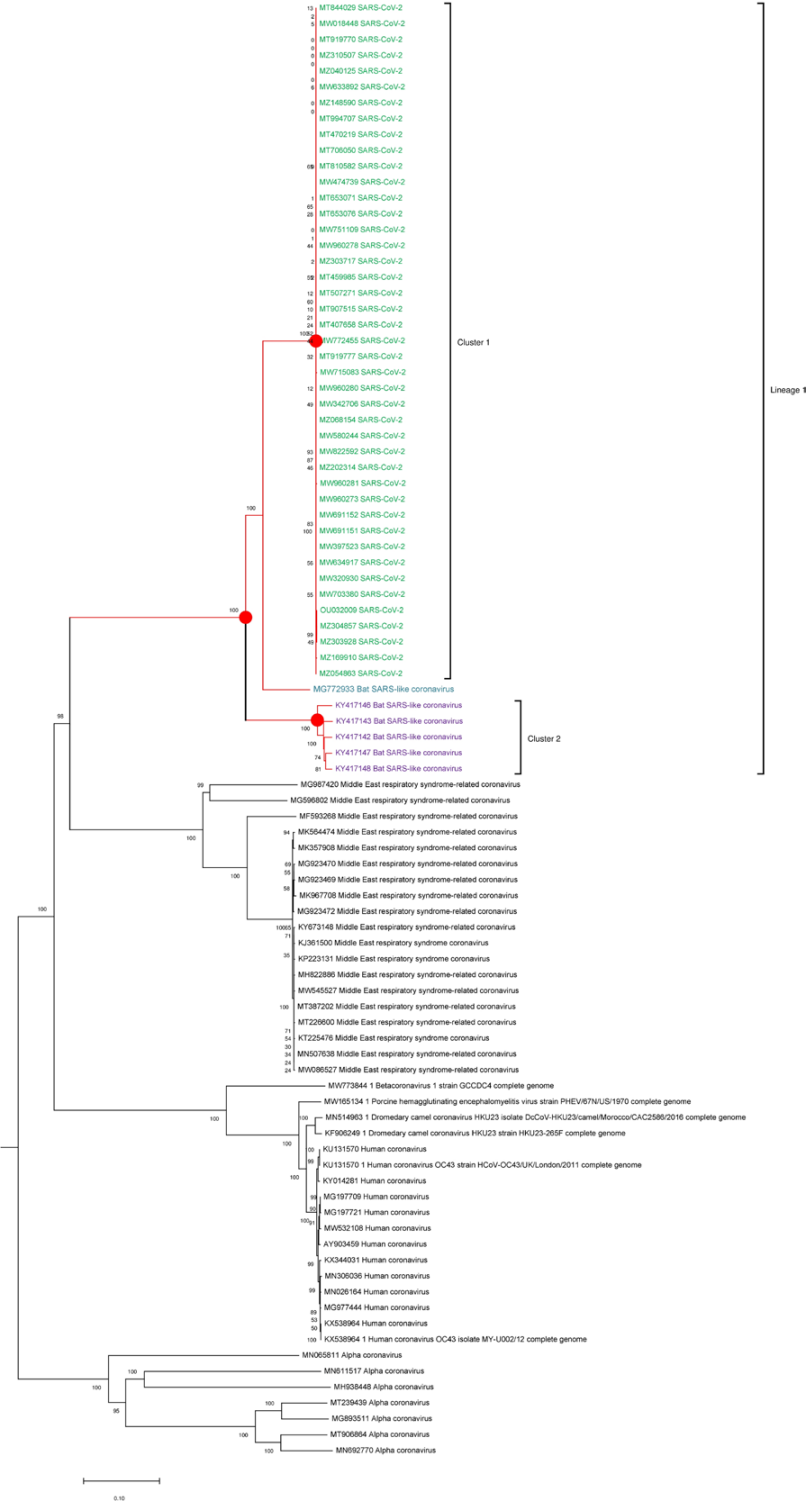
Phylogenetic analysis of severe acute respiratory disease strains. The evolutionary history was inferred using the NJ method. The optimal tree with the sum of branch length = 3.17410000 is shown. The percentage of replicate tree in which the taxa clustered together in the bootstrap test (1000) is shown next to the branches.

Of these 92 isolates, phylogenetic analysis using the NJ method, 49 isolates of respiratory disease with their respective accession numbers were grouped in lineage 1. Furthermore, within lineage 1, which were designated as cluster 1 and cluster 2, respectively, with a bootstrap value of 100 (Fig. 1). Forty-three isolates of SARS-CoV-2, with the exception of 1 BAT-SL-CoV (ACC#MG772933), grouped in cluster 1, which was a distinct cluster but closely related to cluster 2, which contains 5 BAT-SL-CoV. This conformational study clearly revealed that the 43 SARS-CoV-2 complete genome nucleotide sequences were genetically closer to the BAT-SL-CoV (Acc#MG772933), thus confirming its zoonotic transmission.

Formerly based on phylogenetic analysis of the novel 2019 coronavirus, Lu et al^[26]^ investigated 10 genome viral sequences that were obtained from 9 patients (8 complete genomes and 2 individual gene sequences), revealing a sequence distinctiveness of >99%. While nCoV-2019 was meticulously correlated with 2 bat-derived coronaviruses, bat-SL-CoVZC45 and bat-SL-CoVZXC21, collected in 2018 from eastern China, Zhoushan with almost 88% uniqueness. However, they were more distant from SARS-CoV 79% and MERS-CoV 50% separately. This suggested that novel coronavirus-2019 clustered in *the Sarbecovirus* subgenus of the *Beta-coronavirus* genus.

### 1.4. SARS-CoV-2: attachment and entry mechanism

A transmembrane spike glycoprotein (S) plays a significant role in host cell receptor recognition, binding through the receptor-binding domain (RBD), and docking to enter the cell by exploiting human type-2 angiotensin-converting enzyme receptor protein (ACE2).^[27]^ Spike (S) glycoprotein comprises 3 purposeful surface units (S1, S2/S2’) that act contrarily in the course of adherence to host cells. The progression of infection begins when the S1 subunit interacts with human ACE2, where structural changes are persuaded by its entrance into the endosome of the target membrane cell. Subunit (S2) is considered as a fusion protein that supports the virus in the fusion through the cell membrane and appears in 3 main states during the fusion process: (1) prefusion native state, (2) prehairpin intermediate state, and (3) subsequent postfusion hairpin state, while the S2’ subunit is a fusion peptide.^[28–30]^

Moreover, engagement of the target receptor (ACE2) increases viral entrance because of the prominence of suitable conformational modifications for virus and host cell fusion.^[31]^ Several host proteases that persuade further cleavage of the spike (S) glycoprotein to increase virus attachment and fusion, such as transmembrane protease serine (TMPRSS2, TMPRSS4, TMPRSS11a, TMPRSS11D), HAT, trypsin, plasmin, and endosomal cathepsin L, an airway/alveolar cell serine protease specifically articulated on epithelial cells of the respiratory tract.^[28,32–34]^

### 1.5. SARS-CoV-2: transmission and clinical features

The mode of transmission from person to person occurs with close contact with an infected person with or without symptoms before the onset of symptoms sneezes or coughs that produce respiratory droplets to spread the pathogen. When inhaled, these aerosols can be settled in the upper and lower respiratory tract or individuals can also be infected by touching their nose, mouth, and eyes that are exposed to contaminated objects prior.^[35,36]^

The clinical characteristics of novel coronavirus (nCoV) disease range from asymptomatic to acute respiratory distress syndrome (ARDS), which may lead to various organ deterioration. Generally, infection by SARS-CoV-2 begins with symptoms such as fatigue, fever, dyspnea, dry cough, sore throat, chest tightness/pain, headache, myalgia, nausea, vomiting, runny nose, and diarrhea. While progression leads to pneumonia, lung collapse even decreases because of the severe increase in inflammatory cytokines such as IL2, IL7, IL10, IP10, MIP1A, MCP1, GCSF, and TNFα.^[7,37]^ The maturation span for virus in mild cases is ≈3 to 14 days, while compared to severe cases, this can last up to ≈25 to 40 days.^[38,39]^

### 1.6. Immune response to SARS-CoV-2

Innate and adaptive immune systems are involved in the pathogenesis of SARS-CoV-2. The innate immune reaction is known to be the first-line of resistance contrary to viral infection; however, if the protective immune response is impaired, this will result in inflammation in disproportionate and straight death.^[40]^ The host cell receptor (ACE2) is largely articulated in various tissues, although the prime target is reflected in epithelial cells. The bronchial mucosa is confined by mucosal-associated invariant T cells (MAIT) and T (γδ) cells, which react promptly to pathogen attack and activate a cytokine reaction crucial for microbial killing.^[41]^ Inflammation in the lung during the unadorned phase is the leading cause of life-threatening respiratory disorders. Innate inflammation is induced by impaired cells in the lungs, which are mostly facilitated by pro-inflammatory macrophages and granulocytes.^[42]^

A specific adaptive immune response in the course of maturation and nonsevere phase is essential to eradicate the virus and prevent disease development to severity.^[43]^

### 1.7. SARS-CoV-2: prevention

Preventive measures, for now, are extremely important to determine the extent of SARS-CoV-2 infection, such as social distancing, frequent hand washing, using disinfectants, and avoiding touching the mouth, nose, and eyes. Additionally, in cases of viral infection, people should be informed and report about close acquaintances and recent travel history.^[44,45]^

### 1.8. SARS-CoV-2: diagnostic to therapeutic approach

Numerous standards for the diagnosis of infection include molecular based findings (RT-PCR) using oro/nasopharyngeal swabs, bronchoalveolar lavage fluid (BALF) for the viral genes (E, N, S, and ORF), radiological opinion, IgG/IgM.^[6,46,47]^

At present, no targeted antiviral vaccine FDA accredited has been available or to treat SARS-CoV-2 associated infection. In the future, a number of alternatives such as monoclonal antibodies, interferon, viral specific vaccine, and molecule-based viral targeting drugs are the therapeutics that can be inspected but are time-consuming.^[10,48,49]^

To date, various effective therapeutic composites have been recognized against MERS and SARS infections, but the novel 2019 acute respiratory disease has not been confirmed extensively.^[7,19]^ Clinically based treatment options that are supportive as well as effective against (nCoV-19) disease are antiviral/antibacterial, kinase inhibitors, and antiinflammatory agents such as *lopinavir, arbidol, INF-α, ritonavir, favipiravir, chloroquine, oseltamivir, remdesivir, darunavir/cococistat, methylprednisolone, and vermectin*.^[50,51]^

## 2. Chronic Myeloid Leukemia

Chronic myeloid leukemia (CML) is a hematologic clonal malignancy that is produced by hematopoietic stem cells (HSC).^[52]^ CML, a distinctive cytogenetic abnormality, involves the translocation of the Abelson oncogene 1 (ABL1) on chromosome 9, and breakpoint cluster region (BCR) on chromosome 22, t(9;22)(q34;q11.2), ensuing BCR-ABL gene union well-known Philadelphia positive (Ph+) chromosome^[53]^

Between 90% and 96%, Philadelphia positive (Ph+) chromosomes in CML were detected. By altering the (3’ to 5’) segment of the ABL oncogene (9q34) and BCR gene (22q11.2), respectively, the BCR-ABL fusion gene, which encodes a constitutive TK active oncoprotein.^[54,55]^ Existing management has developed in the past and generally comprises the practice of TKIs to constrain the activity of BCR-ABL TK in CML patients triggered by Philadelphia chromosome.^[56,57]^

Individuals with leukemia are believed but not yet known at greater threat and possibly predisposed to novel bat acute respiratory 2019 disease as they are frequently immunosuppressed, have myelosuppression, and have fewer immunoglobulin levels. However, those in the chronic phase, known as chronic myeloid leukemia (CML), rely on BCR-ABL TKIs and are mostly not at a great chance of infection until and unless they do not respond or have poor response to treatment and other multiple comorbidities.^[58,59]^

## 3. Tyrosine Kinase Inhibitors

Tyrosine kinases proteins are a family of enzymes, act as a fundamental moderator in diverse cellular signaling transduction trails through phosphorylation, leading to cell proliferation, differentiation, apoptosis, and metabolism. Approximately 300 kinases exist in every cell. In the progression of malignancies, TKs have been associated with acquired mutation and malignancy, which marks the enzymes vigorous and phosphorylates the downstream cataract.^[60,61]^

Tyrosine kinases (TKs) are a class of small molecules based, rationally designed anti-tumor targeted drugs an eminent antitumor activity, contrary to several tumors comprising chronic myeloid leukemia and gastrointestinal stromal tumors (GIST).^[61]^

Three first-line 3 TKIs (nilotinib, imatinib, and dasatinib) and second-line therapy (ponatinib and bosutinib) are available. In the case of the T3151 mutation, principally ponatinib is specified.^[62]^ In 2001, the leading TK inhibitor used to treat chronic myeloid leukemia (CML) permitted by the Food and Drug Administration (FDA) was the first-generation kinase drug (imatinib), followed by inhibitor therapeutics established in the treatment of advanced-phase CML were mentioned as a second (nilotinib, dasatinib, bosutinib) and third-generation (ponatinib).^[61,62]^ However, TKI treatment based on previous investigations has antiviral, immune-modulator, and endothelium-protective features against SARS and MARS, as well as possibly effective in SARS-CoV-2 infection to inhibit kinase signaling ABL (Abelson) activity and associated pathways to block viral entry.^[63,64]^

A study based on the prevalence and outcome of severe acute respiratory disease 2019 infection in chronic myeloid patients was published by Wang et al.^[65]^ According to the data evaluated based on symptomatic and asymptomatic SARS-CoV-2 infection in patients who actively received BCR-ABL therapy or their response. The optimal response to CML therapy was 1/299 patients (0.3%) compared to those who did not respond to CML treatment was (1/50) with 2% diagnosed with nCoV-19. Consequently, patients who did not achieve an OR (optimal response) to BCR-ABL therapy seemed to have symptomatic SARS-CoV-2 infection.

Here, the probable effective role of TK inhibitor (1) explained that an ideal response to TKI therapy might be related to immune recovery,^[66–68]^ (2) in vitro study of TKI therapy or imatinib as an antiviral against SARS/MERS-CoV disease.^[69–71]^

## 4. Conclusions

The SARS-CoV-2 outbreak has now been considered a universal plague by affecting more than 200 countries, and billions of people have become infected and die due to the novelty of the virus itself. In addition, targeted therapeutic management by avoiding vial spread is challenging. The previous studies have revealed that TKI treatment and their efficacy in SARS and MARS coronaviruses were 3 kinase signaling pathway inhibitors (imatinib, nilotinib, and dasatinib). TKIs are used in targeted therapies for treating CML. Therapy with BCR-ABL TKIs in CML patients so far has been considered to restrain the possibility of novel acute respiratory infection, as well as those who were infected, improved the prognosis. This suggests that TKI treatment as an antiviral agent with other combinations will also enhance the protection, moderate the latent transmission peril, and deliver sufficient kind of management for patients with current novel coronavirus disease (SARS-CoV-2).

## Author contributions

Conceptualization: Asif M and Hussain A

Data curation: Asif M, Amir M and Pushparaj PN.

Formal analysis: Rasool M and Pushparaj PN.

Project administration: Asif M

Writing – original draft: Asif M and Amir M

Writing – review & editing: Rasool M, Pushparaj PN, Achakzai NM and Hussain A
